# The Quality of Infection

**Published:** 1881-07

**Authors:** 


### THE QUALITY OF INFECTION.

The following sensible remarks appear in a little pamphlet by Dr. David Page, of Edinburgh.—(*Mich. Med. News*). Infectious diseases exhibit widely different degrees of severity, but it is well to bear in mind that the slightest and most incipient degree is as intensely infectious as the most alarming and developed, and that the same unrelaxed precautions are required for the one as for the other. Mildness of attack is only a matter of good fortune to the sick person thus attacked, and gives no security whatsoever to the healthy.

Do not forget that infection is a quality of the fever poison, and not altogether one of dose or length of exposure. The mildest case and a moment's exposure may give the infection and the disease in its most malignant form, the issue depending upon the state of the individual and his sanitary surroundings at the time, in other words, *the seeds of infection are always the same, the result depending upon the soil in which they happen to be sown.* I have known some of the worst and most fatal outbreaks of scarlet fever to have been preceded in the affected households by the mildest possible attacks.—*Canada Health Journal.*

